# Genomic Landscape of Vinflunine Response in Metastatic Urothelial Cancer

**DOI:** 10.3390/cancers14020378

**Published:** 2022-01-13

**Authors:** Alejandra Bernardini, Marta Dueñas, María Cruz Martín-Soberon, Carolina Rubio, Cristian Suarez-Cabrera, Raquel Ruiz-Palomares, Ester Munera-Maravilla, Sara Lázaro, Iris Lodewijk, Daniel Rueda, David Gómez-Sánchez, Teresa Alonso-Gordoa, Javier Puente, Álvaro Pinto, Pilar González-Peramato, Carlos Aguado, Mercedes Herrera, Flora López, Victor M. G. Martinez, Lucía Morales, Daniel Castellano, Jesús M. Paramio, Guillermo de Velasco

**Affiliations:** 1 Instituto de Investigación i+12, Hospital University “12 de Octubre”, 28040 Madrid, Spain; Alejandra.Bernardini@externos.ciemat.es (A.B.); marta.duenas@ciemat.es (M.D.); mcms.207@gmail.com (M.C.M.-S.); Carolina.Rubio@externos.ciemat.es (C.R.); cristian.suarez@ciemat.es (C.S.-C.); raquel.ruiz@externos.ciemat.es (R.R.-P.); ester.munera@ciemat.es (E.M.-M.); irisadriana.lodewijk@externos.ciemat.es (I.L.); druedafer.hdoc@gmail.com (D.R.); dagsbio@gmail.com (D.G.-S.); VictorManuel.Garcia@ciemat.es (V.M.G.M.); MariaLucia@externos.ciemat.es (L.M.); cdanicas@hotmail.com (D.C.); 2 Unidad de Oncología Molecular, CIEMAT, 28045 Madrid, Spain; sara.lazaro@ciemat.es; 3 Centro de Investigacion en Red de Cáncer CIBERONC, 28029 Madrid, Spain; 4 Departamento de Oncología Médica, Hospital 12 de Octubre, 28040 Madrid, Spain; herrerajuarezz@gmail.com (M.H.); f17lora_989@hotmail.com (F.L.); 5 Departamento de Oncología Médica, Hospital Ramón y Caja, 28034 Madrid, Spain; talonso@oncologiahrc.com; 6 Departamento de Oncología Médica, Hospital Clínico San Carlos, 28040 Madrid, Spain; javierpuente.hcsc@gmail.com (J.P.); carlos.aguado84@gmail.com (C.A.); 7 Departamento de Oncología Médica, Hospital La Paz, 28046 Madrid, Spain; alvaropintomarin@gmail.com (Á.P.); pilar.gonzalezperamato@uam.es (P.G.-P.)

**Keywords:** urothelial cancer, bladder, vinflunine, biomarkers, immune signatures

## Abstract

**Simple Summary:**

Few metastatic urothelial cancer patients achieve durable clinical benefit with vinflunine. Predictive biomarkers to help to identify better treatment strategies are extremely needed. The objective of this study was to identify molecular differences between extreme responders to vinflunine in urothelial cancer. Genomic and immune markers are potentially useful identifying patients that may achieve greater benefit with vinflunine.

**Abstract:**

Background and Aims: Metastatic urothelial carcinoma (mUC) remains an incurable disease with limited treatment options after platinum-based chemotherapy and immune checkpoint blockade (ICB). Vinflunine has shown a modest increase in overall survival and remains a therapeutic option for chemo- and immunotherapy refractory tumours. However, biomarkers that could identify responding patients to vinflunine and possible alternative therapies after failure to treatment are still missing. In this study, we aimed to identify potential genomic biomarkers of vinflunine response in mUC patient samples and potential management alternatives. Methods: Formalin-fixed paraffin-embedded samples of mUC patients (*n* = 23) from three university hospitals in Spain were used for genomic targeted-sequencing and transcriptome (using the Immune Profile panel by NanoString) analyses. Patients who received vinflunine after platinum-based chemotherapy failure were classified in non-responders (NR: progressive disease ≤ 3 months; *n*= 11) or responders (R: response ≥ 6 months; *n* = 12). Results: Genomic characterization revealed that the most common alteration, *TP53* mutations, had comparable frequency in R (6/12; 50%) and NR (4/11; 36%). Non-synonymous mutations in *KTM2C* (4/12; 33.3%), *PIK3CA* (3/12; 25%) and *ARID2* (3/12; 25%) were predominantly associated with response. No significant difference was observed in tumour mutational burden (TMB) between R and NR patients. The NR tumours showed increased expression of diverse immune-related genes and pathways, including various interferon gamma-related genes. We also identified increased *MAGEA4* expression as a potential biomarker of non-responding tumours to vinflunine treatment. Conclusions: Our data may help to identify potential genomic biomarkers of response to vinflunine. Moreover, tumours refractory to vinflunine showed immune signatures potentially associated with response to ICB. Extensive validation studies, including longitudinal series, are needed to corroborate these findings.

## 1. Introduction

Metastatic urothelial cancer (mUC) is an aggressive disease with a high mortality rate, causing nearly 200,000 deaths in 2018 [1]. Platinum-based combination chemotherapy (CT) has been the standard regimen in first-line treatment for advanced UC during the last three decades and provided outcomes leading to a median overall survival (OS) between 12–15 months and 5-year survival of 13–15% [2]. Despite this clear benefit in response, most patients eventually become resistant and progress to metastatic disease [3]. 

Immunotherapy based on ICB has become a new standard of care after CT progression [4,5] as well as maintenance in advanced UC patients whose disease has not progressed during first-line CT [6]. Nonetheless, CT may be indicated for those who are not candidates for ICB and for those patients who progress during or after immunotherapy [4,7]. Regardless, therapeutic options are significantly reduced upon the failure of current treatments [8]. 

ICB and platinum-based CT offer a long period of control of disease only in a minority of patients. Consequently, other therapeutic approaches are needed. Vinflunine, a microtubule inhibitor approved by European Regulatory Agencies in 2009 [9,10], has been widely used in Europe after platinum-based CT progression. Unfortunately, only a subset of patients derives substantial benefit from vinflunine treatment (median progression-free survival (PFS) ≈ 3 months) [11]. Complete responses to this therapy are rare but some patients significantly benefit, showing durable partial response (PR) and/or extended disease control, even if not meeting the criteria for a PR [12]. Nonetheless, there are no reliable biomarkers that may explain or predict which patients may benefit from the different available treatments, including vinflunine. 

Although extreme responders to vinflunine account for less than 10% of the patients, their characterization may provide useful information about the possible therapeutic options after treatment failure. In this work, we characterize the genomic landscape of extreme responder (R) and non-responder (NR) patients to vinflunine to seek molecular determinants of response. We also characterized their immune transcriptomic profiles in order to determine whether ICB could represent a suitable alternative or potential approach after vinflunine failure in mUC. 

## 2. Methods

### 2.1. Patients

Inclusion criteria were patients older than 18 years, with diagnosis of advanced UC refractory to first-line platinum-based CT recruited from university hospitals “12 de Octubre”, “La Paz” and “Ramón y Cajal” in Madrid, treated with vinflunine as a monotherapy regimen (dose range 250–320 mg/m^2^, every 3 weeks), and finished treatment by 2016. The ethics committee from “12 de Octubre” Hospital approved the study (ref. 17/094) and notified to other institutions involved. Discrimination of response in patients was based on RECIST v 1.0 criteria. For this study, we defined response R as those patients showing clinical benefit for at least 6 months, including PR or stable disease (SD) with any tumour shrinkage (no growth). NR included patients showing progressive disease (PD) within the first 3 months of therapy (usually at first restaging), without marked toxicity leading to treatment discontinuation. This arbitrary division was made based on the median PFS in the pivotal trial with vinflunine [9]. Formalin-fixed paraffin-embedded (FFPE) tissue samples with tumour components higher than 75% were used for genomic and/or immune transcriptome profiling.

### 2.2. Next Generation Sequencing and Variant Calling

DNA was extracted from FFPE tissue sections using GeneRead DNA FFPE Kit (Cat.No.56404, QIAGEN). Sequencing-ready libraries were prepared using the Human Comprehensive Cancer Panel (Cat.No.DHS-3501Z, QIAGEN). Library construction and target enrichment were performed following the manufacturer’s indications for FFPE samples using 100 ng of DNA, determined by Qubit 4 (Thermo Fisher Scientific, Waltham, MA, USA).

Sequencing was performed on an Illumina NextSeq 500 sequencer (Illumina, Nextera XT v2 adapter sample index system). Each DNA library was diluted to 4 nM, after which they were pooled and the final concentration of DNA multiplex sample loaded on sequencer was 1.65 pM as indicated by manufacturer’s instructions (Illumina). The libraries were subjected to paired-end sequencing with a read-length of 2 × 150 bp. Tumour material was sequenced to average depth coverage of 440× and more than 7 million reads per sample were obtained.

Sequence alignment, variant calling and annotation were performed by smCounter algorithm for QIAseq targeted DNA panels [13] in QIAGEN’s QIAseq targeted sequencing data analysis portal (https://geneglobe.qiagen.com/us/analyze (accessed on October 2020). Variants were filtered according to allelic frequency (variants with frequency lower than 0.1 were discarded) and population frequency (variants present in population in a frequency at least 0.001 were discarded) to discard potential artefacts and polymorphisms, respectively. All loss-of-function alterations were considered deleterious, including deletions and frameshift or splice site alterations. For non-synonymous mutations, deleterious status was determined by manual review annotation of oncogenicity by OncoKB [14] or recurrent mutations in the Catalogue of Somatic Mutations in Cancer (COSMIC) [15] and consensus effect predictor indicating deleterious mutation by Varsome [16]. KEGG database was used to look for altered pathways in each of both groups (R and NR) [17].

All single nucleotide changes obtained were used to perform an analysis of mutational signatures, which was performed with the Mutational Signatures R Package [18].

### 2.3. Gene Expression Profiles

Total RNA was isolated from FFPE samples using the miRNeasy Mini Kit (QIAGEN) and DNA was eliminated (RNase-Free DNase Set (QIAGEN)). PanCancer Immune Profiling Panel (Cat XT-CSO-HIP1-12) from NanoString technology (Seattle, WA, USA) was used to analyze the expression of genes covering both the adaptive and innate immune response and for comprehensive profiling of immune response categories. Reporter CodeSet provided by Nanostring was mixed with 250 ng of total RNA from each FFPE sample and incubated at 67 °C for 20 h according to the manufacturers’ standard protocol. Data were obtained using nCounter FLEX Analysis System and analyzed by nSolver Analysis Software 4.0 and nCounter Advanced Analysis 2.0.

### 2.4. Immunohistochemistry and Real Time Quantitative PCR for MAGEA4

For immunohistochemistry (IHC), FFPE sections (4 µm thick) were deparaffinized, and antigen retrieval was performed with citric acid buffer (pH 6), using a pressure cooker (Dako, Agilent Technologies). Endogenous peroxidase was inhibited by incubation with hydrogen peroxide (0.3% in methanol, 15 min). Non-specific epitopes were blocked with 10% horse serum, after which the sections were incubated with the MAGE A4 antibody (HPA021942, Merck) diluted (1/1000) in 10% horse serum followed by biotin-labelled secondary antibody (711-065-152 Jackson Immunores. diluted 1/1000). The signal was amplified using avidin-peroxidase (VECTASTAIN^®^Elite^®^ ABC Kit; Vector Laboratories, Burlingame, CA, USA) and peroxidase was exposed using diaminobenzidine as a substrate (DAB Substrate Kit; Vector Laboratories), according to the manufacturer’s instructions. Immunostaining was calculated by double blind analyses from 0 to 4 scoring at least 6 (20×) fields per slide. 

For real time quantitative PCR (RT-qPCR), total RNA was isolated as previously mentioned. Reverse transcription was performed using the Omniscript RT Kit (QIAGEN) following the manufacturer’s instructions. Specific primers for *MAGEA4* (5′-TTTCTTCAAACAGAGTGAA-3′) and *GUSB* (5′-CTTCTGATACTTCTTATAC-3′) were used. The RT-qPCR was performed in a 7500 Fast Real Time PCR System using Power SYBR GREEN PCR master mix [Applied Biosystems]. 1 μL of cDNA template was used with the specific amplification primers for *MAGEA4* (Forward 5′-AGGGAGTCTGAGCATGAGTTG-3′ and Reverse 5′-CACAGGGCTGTTAGATGCAC-3′) and for *GUSB* (Forward 5′-CCTGTGACCTTTGTGAGCAA-3′ and Reverse 5′-AACAGATCACATCCACATACGG-3′). Melting curves were performed to verify the specificity and the absence of primer dimers. Reaction efficiency was calculated for each primer combination, and *GUSB* was used as a reference gene for normalization.

### 2.5. Statistical Analysis

Differences between groups were evaluated by t-test. Fisher’s exact test was used to evaluate differences in contingency tables. A *p*-value ≤ 0.05 was considered statistically significant. In gene expression analysis, the *p*-value was calculated using the Benjamini–Yuketeli test and a threshold of BY *p*-value of 0.5 was given.

### 2.6. Data Availability

The datasets generated during and/or analyzed during the current study are available from the corresponding author on reasonable request.

## 3. Results 

### 3.1. Clinical Characteristics

**Figure 1:**
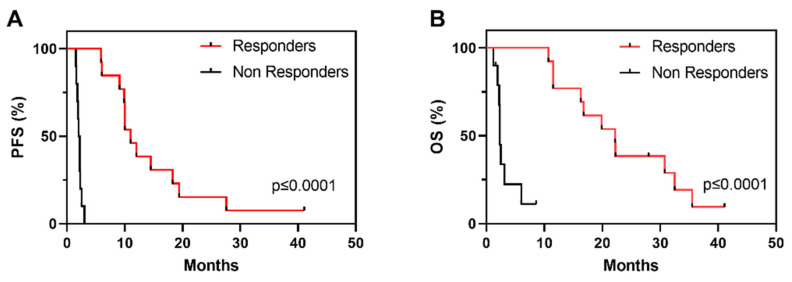
Kaplan–Meier survival curves showing progression free survival (**A**) (PFS) and overall survival ((**B**), OS) in R and NR patients in the study series to vinflunine treatment; *p*-Values provided by log rank test).

Demographics and clinicopathological characteristics as well as OS and PFS of the cohort are described in Table 1. Of the overall 23 patients, 12 patients were classified as R, showing PR or SD with tumour shrinkage, and 11 patients were classified as NR, showing disease progression within the first 3 months of vinflunine treatment (Table 1). PFS clearly identifies two populations of R and NR to vinflunine (Figure 1A), which also correspond to increased overall survival (OS) (Figure 1B).

### 3.2. Somatic Genetic Alterations 

**Figure 2:**
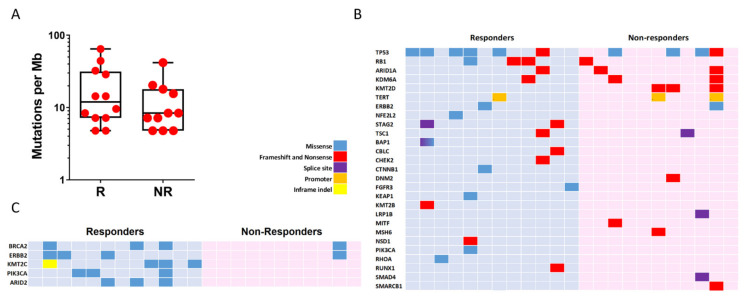
Genomic alterations in mUC samples. (**A**) Count of non-synonymous mutations per Mb in responder (R) and non-responder (NR) patients. (**B**) Mutation plot showing oncogenic variants annotated in OncoKB, recurrent in COSMIC or indicated deleterious by Varsome. (**C**) Mutation plot showing genes with two or more alterations when comparing responders and non-responders group. Gene alterations for B and C are annotated according to the colour panel.

We determined mutational landscape of mUC by NGS using primary tumour samples. We found that the two groups (R and NR) showed similar mutation burden (11.95 vs. 8.37, respectively, *p*-value = 0.28; Figure 2A). All observed somatic mutations and short insertion/deletions are shown in Supplementary Figure S1, and those previously associated with cancer are shown in Figure 2B. The mutated genes and their mutation frequency were similar to those reported by TCGA, except for *MSH6* and *KMT2B* genes, which showed reduced frequency in our series (26% and 13% in TCGA vs. 2.7% and 6% in our series, respectively). *TP53* (10/23; 43.4%) and *KMT2D* (8/23; 34.8%) were the most commonly altered genes in our series, without significant differences in R and NR groups (*TP53*, 6/12, 50% in R and 3/11, 27% in NR, *p*-value = 0.68; *KMT2D*, 0 in R and 27% in NR, *p*-value > 0.69). We observed that the two groups could not be discriminated using alterations in any cancer-associated gene. However, considering all mutations (not only those previously reported as potential oncogenic drivers), those affecting *KTM2C* (4/12; 33.3%, R vs. 0 in NR *p*-value = 0.09), *PIK3CA* (3/12; 25%, R vs. 0 in NR, *p*-value = 0.22) and *ARID2* (3/12; 25%, R vs. 0 in NR, *p*-value = 0.22) were only observed in R patients (Figure 2C). In addition, we observed that the R group showed a partial enrichment in mutations affecting *BRCA2* (25% vs. 9%, R vs. NR, *p*-value = 0.59) and *ERBB2* (25% vs. 9%, R vs. NR, *p*-value = 0.59) (Figure 2C). No altered drivers, gene sets or pathways were found to segregate the two groups. However, the R group displayed a substantial enrichment in the RAP1 signalling pathway (hsa04015 KEGG pathway) affecting *CTNNB1*, *PIK3CA*, *RHOA* and *FGFR3* whereas no genes involved in this pathway were mutated in the NR group (Figure S2). This observation could be relevant as this pathway may exert oncogenic and tumour suppressor functions involving cell adhesion, cell–cell junction formation and cell polarity, and includes different genes that can be targeted by specific inhibitors.

We also analyzed the distribution of mutational signatures in the R and NR groups. The optimal contribution of COSMIC signatures to reconstruct 96 mutational profiles revealed no different patterns of mutational signatures between the two tumour groups (Figure S3). 

### 3.3. Tumour Immune Expression Is Associated with Differences in Vinflunine Efficacy

The response to ICB therapies in UC is associated with the presence of specific immune gene signatures and the presence of specific immune cell infiltrates. To monitor whether ICB therapy could be a possible therapeutic alternative to vinflunine or a potential treatment after vinflunine progression, we performed immune gene profiling using the nCounter PanCancer Immune Profiling Panel in primary tumour samples [19].

**Figure 3:**
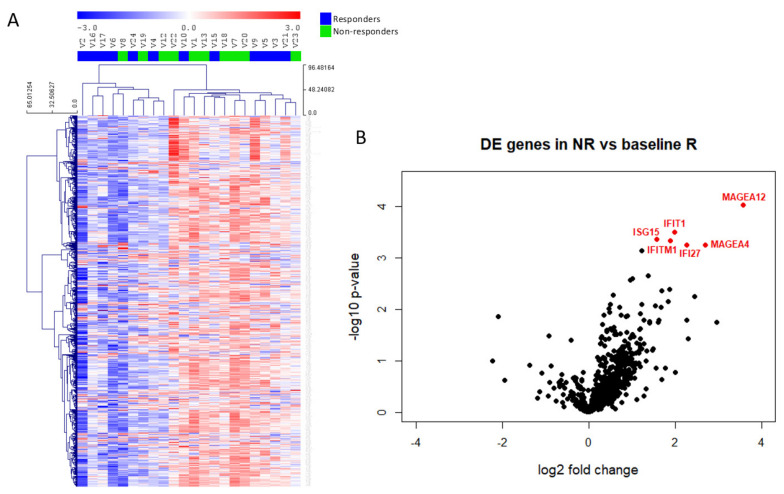
Immune gene expression in mUC samples. (**A**) Heatmap of the normalized gene expression generated via unsupervised clustering. Red indicates high expression; blue indicates low expression (log2 scale). (**B**) Volcano plot displaying each gene’s −log10 (*p*-value) and log2 fold change between non-responders (NR) and responders (R). Statistically significant expressed genes (below the given *p*-value threshold [<0.05]) are marked in red.

The unsupervised clustering of all samples according to immune gene profiling revealed no significant DEGs between R and NR samples (Figure 3A), whereas a supervised classification revealed that only 6 genes discriminated the two groups (*p*-value < 0.001 and Benjamini–Yekutieli FDR < 0.5). We observed increased expression of *MAGEA12*, *IFIT1*, *ISG15*, *IFITM1*, *IFI27* and *MAGEA4* in patients showing no clinical benefit to vinflunine treatment (Figure 3B). Gene expression values and ratios as well as statistical information are shown in Table 2. In addition to the above commented genes, we also studied potential differences in several relative signature scores among R and NR (Table 3 and Figure S5). These signature scores were calculated as the first principal component of the pathway genes normalized expression. Although no significant differences were obtained between scores in R and NR samples (Figure S5), there was a trend that indicates that NR might have more activated pathways related to immune response. 

The *IFIT1*, *ISG15*, *IFITM1* and *IFI27* genes are related to activation of the interferon gamma (IFNγ) response, suggesting possible inflammatory responses in NR tumour samples. The expression of these genes showed a significant correlation, both in R and in NR tumour samples. *MAGE* gene family did not show correlation with interferon related genes (Figure S4), but a positive correlation was observed between *MAGEA4* and *MAGEA12* in R and NR tumour groups (Figure S4).

**Figure 4:**
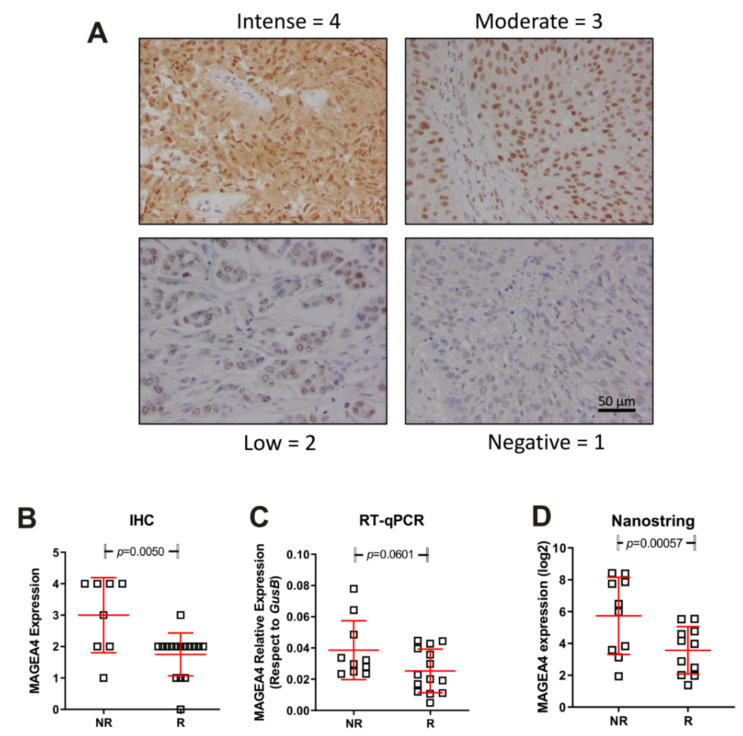
Expression of MAGEA4 in responders (R) and non-responders (NR). (**A**) Representative examples corresponding to the different IHC scores. (**B**–**D**) Summary of the MAGEA4 expression measured by IHC (**B**) mRNA expression by probe hybridization in nCounter Nanostring platform (**D**), mRNA expression by RT-qPCR (**C**). *p*-Values were estimated by un-paired T test.

We next validated the above commented data regarding high expression of *MAGEA4* gene in NR patients. To this, we used an independent second series of tumour samples (anonymized mUC patients treated with vinflunine R = 14; NR = 10 provided from Biobank of University Hospital “12 de Octubre”). We performed Nanostring, immunohistochemistry and RT-qPCR studies. The results confirmed our previous observation (Figure 4) and strongly suggested that MAGEA4 expression could help to identify mUC patients at high predisposition of no response to vinflunine treatment.

In summary, our results indicate that patients with low probability of benefit to vinflunine treatment display increased immune infiltrate and can be characterized by increased expression of markers such as *MAGE A4*.

## 4. Discussion

Biomarkers of response to available therapy are not robust enough to select strategies upfront for mUC patients. The current standard of care remains platinum-based CT followed by ICB maintenance, a non-curative strategy. In addition, not all patients achieve clinical benefit and specific molecular characterization is not currently useful to either predict resistance or prevent unnecessary toxicity. Strikingly, some patients could still benefit from available treatments but the efforts to understand these drugs are tremendously limited. In Europe, vinflunine is still the standard of care after platinum and ICB, and may provide long term benefit to a minority of patients. Herein, we decipher genomic characterization, including mutational and transcriptomic signatures to discriminate the patients that may benefit from vinflunine. 

The diversity and complexity of somatic mutational processes underlying carcinogenesis in UC has been largely described. Mutational status and molecular signatures have revealed insight into cisplatin response in UC [20,21]. Most UC mutations are clonal but the TMB, largely associated with response to immunotherapy, is neither a reliable biomarker to select treatment nor a response to vinflunine. 

Currently, the role of tumour immune infiltrate remains unclear in UC. Tumour infiltrating lymphocytes (TILs) have been associated with different outcomes in mUC. Specifically, in muscle-invasive UC, CD8+ TILs have been associated with better survival in localized tumours [22] as well as in metastatic tumours treated with platinum-based CT [23]. *IFI27* has been shown to be upregulated by IFNγ [24]. *ISG15* potentiates IFNγ immunity and lymphocyte production [25]. *IFIT* genes are induced by IFNα treatment and to lesser extent by exposure to IFNγ. Nevertheless, IFITM proteins are generally induced to greater levels than IFIT proteins by IFNγ [26]. These upregulated genes could be promoting an enhanced inflammatory response in NR patients, making them sustainable to ICB therapy. Collectively, expression of the signature scores, including chemokines and cytokines, was different among R and NR, which supports our hypothesis that immune infiltration may also define treatment choice. 

Our mRNA expression analysis identified two genes that showed significantly higher expression in the NR group, indicating their potential use as predictive biomarkers: *MAGEA12* and *MAGEA4*. In addition, the high expression of *MAGEA4* associated with low response indicated that this high expression could represent an indication to select vinflunine as a therapeutic option. Nevertheless, it was shown that enhanced expression of CT Antigens makes cells more susceptible to CT antigen-specific cytotoxic T cells mediated killing [27], making these patients potential candidates for ICB therapy. The high expression of MAGEA associated with progression could be relevant for treatment selection, since increased expression of a subcluster of MAGE-A cancer-germline antigens has been shown to predict resistance specific to CTLA-4, but not PD-1, blockade, and its association with autophagy suppression implicates the role of autophagy in regulating primary resistance to anti-CTLA-4 therapy. Presently, there is an ongoing clinical trial testing the effectiveness of anti-MAGE-A3/12 in TCR-gene engineered lymphocytes in metastatic cancer patients (NCT01273181).

The study has several limitations. Despite the careful selection of patients, the sample size is small. Even though patients with similar clinical and molecular characteristics are compared, there is substantial unexplained variation in patient outcomes, which makes it extremely difficult to drive robust conclusions. For example, recent data have shown that there may be differences observed during tumorigenesis and clinical outcomes based on sex-specific gene regulatory networks [28]. The main limitation of the study is that the observations are hypothesis-generating, and thus are not ready to be used for clinical decision making. While additional studies are required, we advocate continuing studying drugs and predictors of response for agents, which have already shown efficacy in a subgroup of patients. Novel tools, novel designs and faster personalized strategies are needed to avoid the standard low process to approve drugs. This attempt may offer new opportunities for patients using available and/or off-patent drugs. 

## Figures and Tables

**Table 1:** Characteristics of patients (*n* = 23).

	Responders	Non-Responders (*n* = 11)	Global	*p* Value
	(*n* = 12)		(*n* = 23)	
Male (%)/Female (%)	11 (19%)/1 (9%)	8 (72%)/3 (28%)	19 (82%)/4 (18%)	0.23
Age media (SD)	67 (9.2)	66 (11.4)	66	0.57
Smokers	8 (66%)	7 (63%)	15 (65%)	0.54
Urothelial carcinoma	12 (100%)	11 (100)	100 (100%)	
1 Line				
0	6 (50%)	5 (45%)	11 (48%)	
1	6 (50%)	5 (45%)	11 (48%)	
2	0	1 (10%)	1 (4%)	0.56
Prior platinum based CT				
Cisplatin	10 (83%)	5 (45%)	15 (65%)	
Carboplatin	2 (17%)	6 (55%)	8 (35%)	0.06
Response to platinum based CT				
Partial response	1 (8%)	5 (45%)	6 (26%)	
Stable disease	6 (50%)	5 (45%)	11 (48%)	
Progressive disease	5 (42%)	1 (9%)	6 (26%)	0.06
Number of line for vinflunine				
2	9 (81%)	10 (90%)	19 (83%)	
3	1 (8%)	1 (9%)	2 (8.6%)	
4	2 (16%)	0	2 (8.6%)	0.36
Location of metastases				
Lung	5 (42%)	4 (36%)	9 (39%)	0.79
Lymph nodes	9 (81%)	7 (27%)	16 (69%)	0.55
Liver	1 (8%)	3 (45%)	4 (17%)	0.23
Bone	4 (36%)	5 (27/)	9 (39%)	0.52
Median PFS (months) of vinflunine treatment (CI 95%)	10 (8.7–11.5)	2.2 (1.8–2.5)	6 (0–15.5)	0.02
Median OS (months) of vinflunine treatment	19.9 (10.7–29.0)	2.5 (2.1–2.8)	11.5 (3.4–19.5)	0.06

PFS: progression-free survival; OS: overall survival; CI: confidence interval.

**Table 2:** Differentially expressed genes in NR vs. baseline R.

Gene	Log2 Fold Change	Std Error (log2)	Lower Confidence Limit (log2)	Upper Confidence Limit (log2)	*p*-Value	BY.*p*-Value	Gene.Sets
*MAGEA12-*	3.58	0.726	2.16	5	0.0000927	0.485	CT Antigen
*IFIT1-*	1.99	0.458	1.09	2.89	0.000311	0.497	Chemokines
*ISG15-*	1.58	0.375	0.84	2.31	0.000442	0.497	
*IFITM1*	1.89	0.452	1	2.77	0.000464	0.497	Regulation
*IFI27-*	2.27	0.555	1.19	3.36	0.000557	0.497	Chemokines
*MAGEA4-*	2.69	0.652	1.41	3.97	0.00057	0.497	CT Antigen

CT antigen: cancer testis antigen.

**Table 3:** Enrichment in Immune-related pathways between R and NR Patients.

Immune Pathway	Signature Scores (NR/R)
Regulation	3.52
Chemokines	3.22
Cytokines	2.06
Cell Functions	1.94
T-Cell Functions	1.83
TNF Superfamily	1.59
Antigen Processing	1.43
Pathogen Defense	1.40
Interleukins	1.30
Adhesion	1.29

## Data Availability

As per individual request.

## Supplementary Materials

The following supporting information can be downloaded at: https://www.mdpi.com/article/10.3390/cancers14020378/s1. Figure S1. Genomic alterations in responder and non-responder mUC patients to vinflunine. Mutation plot showing individual mUC samples on the x axis. Gene alterations are annotated according to the colour panel at right side of image. The frequency of appearance of the mutation in all, responder and non-responder patients, is plotted on the right panel. Mutation burdens and type of base-pair substitution and indels are displayed in the top and bottom panel, respectively. Figure S2. RAP1 signalling pathway enrichment. KEGG pathway (hsa04015) showing RAP1 signalling pathway as appears in https://www.genome.jp/kegg-bin/show_pathway?hsa04015 (accessed on October 2021). Genes mutated in responders are marked in red. Figure S3. COSMIC mutational signatures associated with alterations in mUC samples. Signatures of mutational process were calculated by the R package MutationalPatterns. Top panel shows the optimal contribution of COSMIC signatures to reconstruct 96 mutational profiles of each sample, including just signatures of optimal contribution. Bottom panel shows the heatmap and sample cluster tree of the relative contribution of COSMIC signatures to reconstruct 96 mutational profiles in each sample. NRV: non-responders to vinflunine. RV: responders to vinflunine. Figure S4. Gene expression correlation plot. Pairwise co-expression of the six differentially expressed genes. Responders’ data are in orange and non-responders’ data are in grey. In the top right fields is indicated numerically the overall correlation of gene expression as the Pearson correlation coefficient and corresponding *p*-value for each pair of genes, also the Pearson values of correlation of gene expression within each group is shown. The diagonal element shows the univariate expression distribution of each gene. In the lower left fields is expressed graphically the correlation of gene expression for each pair of genes plotting the expression values and separating the groups by colour. Figure S5. Signature scores associated with immune regulation in mUC samples between responders and non-responders. Pathway scores are calculated in each covariate from the expression value of each gene associated to the pathway.
