# Supplementary material for: Genomic Landscape of Vinflunine Response in Metastatic Urothelial Cancer
Source: Cancers (Basel). 2022 Jan 13;14(2):378. doi: 10.3390/cancers14020378 (PMC8773703; doi:10.3390/cancers14020378)
Supplement: Supplementary file 1 [file cancers-14-00378-s001.zip › cancers-1510799-supplementary.pdf]

Supplementary Information to

Genomic landscape of vinflunine response in metastatic urothelial cancer

by

Alejandra Bernardini[1,2,3], Marta Dueñas[1,2,3], M. Cruz Martín[1,4], Carolina Rubio[1,2,3], Cristian Suarez-Cabrera [1,2,3] Raquel Ruiz-Palomares[1,2], Ester Munera-Maravilla[1,2,3], Sara Lázaro[2], Iris Lodewijk[1,2,3], Daniel Rueda[1], David Gómez[1], Teresa Alonso[4], Javier Puente[6], Álvaro Pinto[7], Pilar González-Peramato[7], Carlos Aguado[6], Mercedes Herrera[4], Flora López[4], Victor M G Martinez[1,2,3], Lucía Morales[1,2], Daniel Castellano[1,3,4],Jesús M Paramio[1,2,3*], Guillermo de Velasco[1,4*]

Authors Affiliations

1. Instituto de Investigación i+12. Hospital Univ. "12 de Octubre". Madrid 28040 Spain
2. Unidad de Oncología Molecular, Departamento de Investigación Básica, CIEMAT. Madrid 28045 Spain
3. Centro de Investigacion en Red de Cáncer CIBERONC
4. Departamento de Oncología Médica, Hospital 12 de Octubre.
5. Departamento de Oncología Médica, Hospital Ramón y Cajal
6. Departamento de Oncología Médica, Hospital Clínico San Carlos
7. Departamento de Oncología Médica, Hospital La Paz

This file contains Supplementary Figures S1 to S5

Supplementary information

Figure S1. Genomic alterations in responder and non-responder mUC patients to vinflunine. Mutation plot showing individual mUC samples on the x axis. Gene alterations are annotated according to the colour panel at right side of image. The frequency of appearance of the mutation in all, responder and non-responder patients, is plotted on the right panel. Mutation burdens and type of base-pair substitution and indels are displayed in the top and bottom panel, respectively.

Figure S2. RAP1 signalling pathway enrichment. KEGG pathway (hsa04015) showing RAP1 signalling pathway as appears in https://www.genome.jp/kegg-bin/show_pathway?hsa04015. Genes mutated in responders are marked in red.

Figure S3. COSMIC mutational signatures associated with alterations in mUC samples. Signatures of mutational process were calculated by the R package MutationalPatterns. Top panel shows the optimal contribution of COSMIC signatures to reconstruct 96 mutational profiles of each sample, including just signatures of optimal contribution. Bottom panel shows the heatmap and sample cluster tree of the relative contribution of COSMIC signatures to reconstruct 96 mutational profiles in each sample. NRV: non-responders to vinflunine. RV: responders to vinflunine.

Figure S4. Gene expression correlation plot. Pairwise co-expression of the 6 differentially expressed genes. Responders data are in orange and non-responders data are in grey. In the top right fields is indicated numerically the overall correlation of gene expression as the Pearson correlation coefficient and corresponding p-value for each pair of genes, also the Pearson values of correlation of gene expression within each group is shown. The diagonal element shows the univariate expression distribution of each gene. In the lower left fields is expressed graphically the correlation of gene expression for each pair of genes plotting the expression values and separating the groups by colour.

Figure S5. Signature scores associated with immune regulation in mUC samples between responders and non-responders. Pathway scores are calculated in each covariate from the expression value of each gene associated to the pathway.

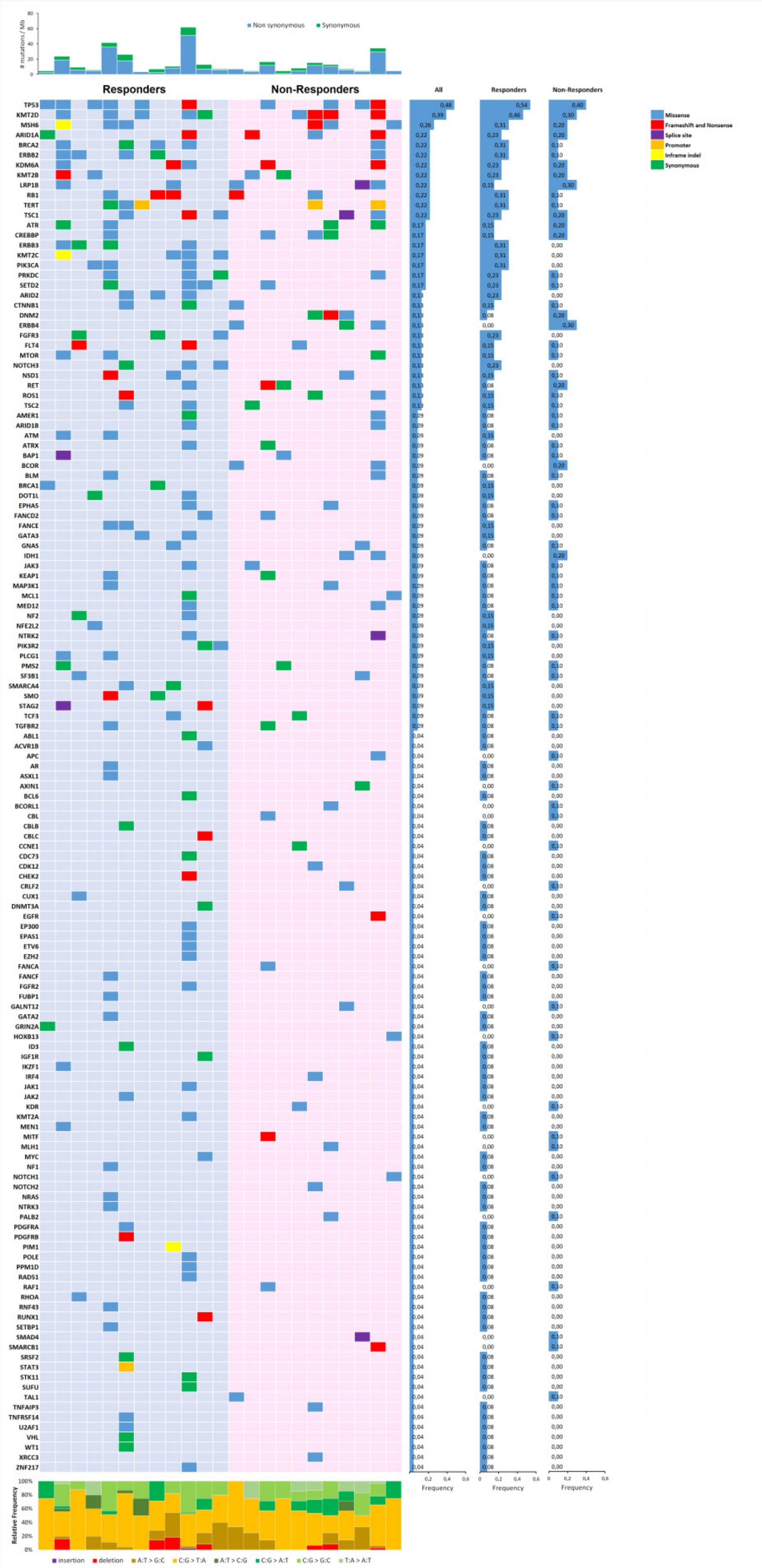

Figure S1

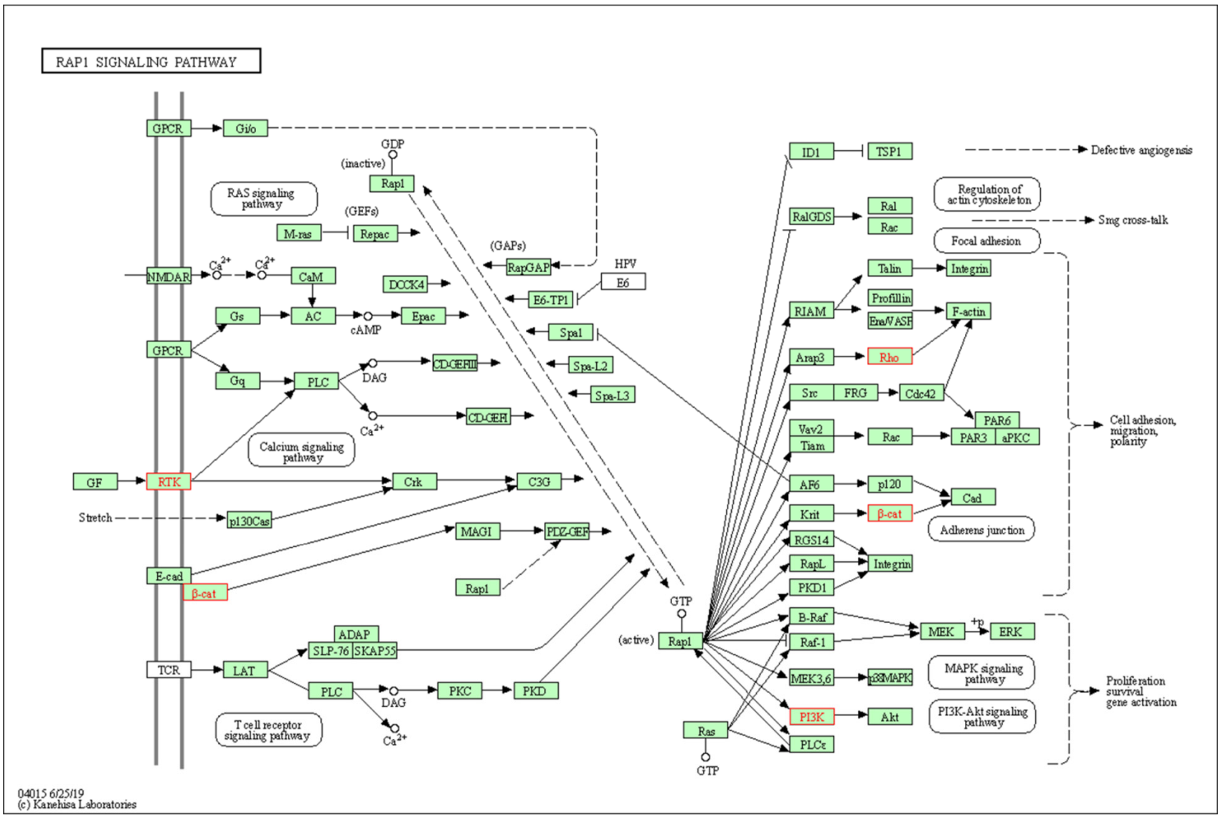
RAP1 SIGNALING PATHWAY
GPCR
Gi/o
GDP
(inactive)
Rap1
RAS signaling pathway
(GEFs)
M-ras
Repac
(GAPs)
RapGAP
HPV
E6
NMDAR
CaM
DOCK4
E6-TP1
Gs
AC
cAMP
Epac
Spa1
GPCR
Gq
PLC
DAG
Spa-L2
Spa-L3
Calcium signaling pathway
GF
RTK
Crk
C3G
Stretch
p130Cas
MAGI
PDZ-GEF
E-cad
β-cat
Rap1
GTP
(active)
ADAP
SLP-76
SKAP55
TCR
LAT
PLC
PKC
PKD
T cell receptor signaling pathway
Ras
GTP
04015 6/25/19
(c) Kanehisa Laboratories
ID1
TSP1
Defective angiogensis
Regulation of actin cytoskeleton
RalGDS
Ral
Rac
Smg cross-talk
Focal adhesion
Talin
Integrin
RIAM
Profillin
Ena/VASP
F-actin
Arap3
Rho
Src
FRG
Cdc42
PAR6
Vav2
Tiam
Rac
PAR3
aPKC
Cell adhesion, migration, polarity
AF6
p120
Cad
Krit
β-cat
Adherens junction
RGS14
RapL
Integrin
PKD1
B-Raf
Raf-1
MEK
+p
ERK
MEK3,6
p38MAPK
MAPK signaling pathway
PI3K
Akt
PI3K-Akt signaling pathway
PLCε
Proliferation survival gene activation

Figure S2

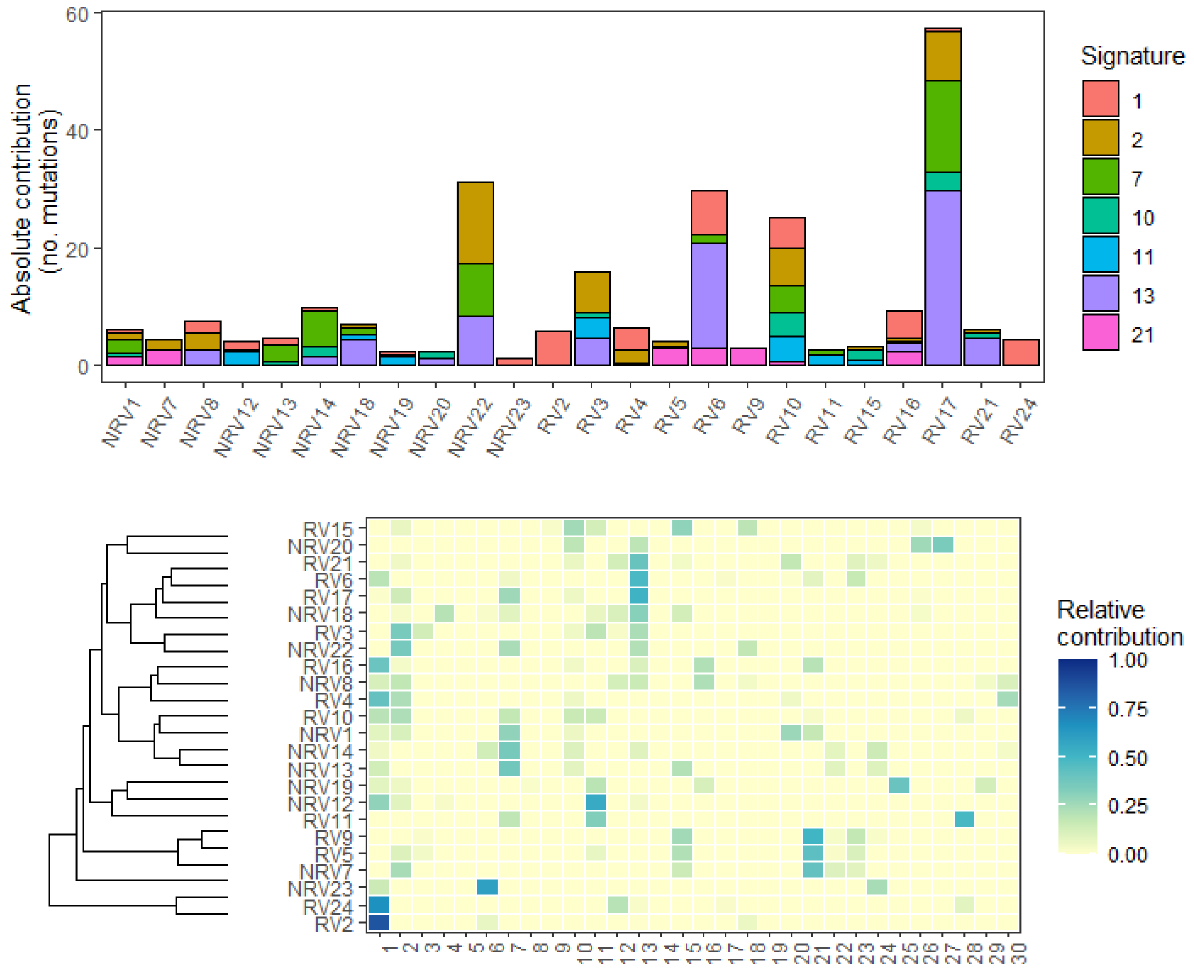

Absolute contribution (no. mutations)
60
40
20
0
Signature
1
2
7
10
11
13
21
NRV1
NRV7
NRV8
NRV12
NRV13
NRV14
NRV18
NRV19
NRV20
NRV22
NRV23
RV2
RV3
RV4
RV5
RV6
RV9
RV10
RV11
RV15
RV16
RV17
RV21
RV24
RV15
NRV20
RV21
RV6
RV17
NRV18
RV3
NRV22
RV16
NRV8
RV4
RV10
NRV1
NRV14
NRV13
NRV19
NRV12
RV11
RV9
RV5
NRV7
NRV23
RV24
RV2
1 2 3 4 5 6 7 8 9 10 11 12 13 14 15 16 17 18 19 20 21 22 23 24 25 26 27 28 29 30
Relative contribution
1.00
0.75
0.50
0.25
0.00

Figure S3

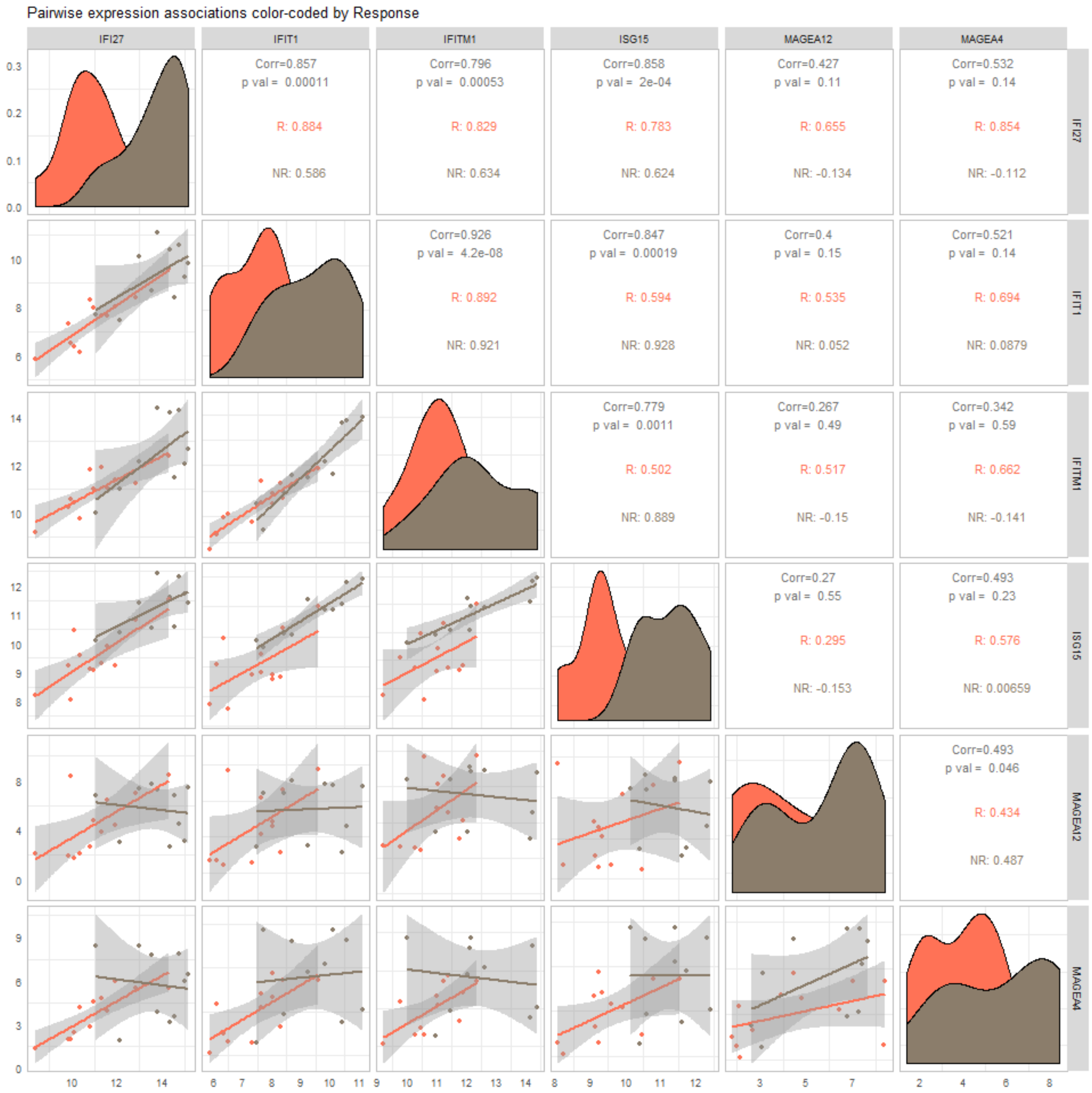
Pairwise expression associations color-coded by Response
IFI27
IFIT1
IFITM1
ISG15
MAGEA12
MAGEA4
Corr=0.857
p val = 0.00011
R: 0.884
NR: 0.586
Corr=0.796
p val = 0.00053
R: 0.829
NR: 0.634
Corr=0.858
p val = 2e-04
R: 0.783
NR: 0.624
Corr=0.427
p val = 0.11
R: 0.655
NR: -0.134
Corr=0.532
p val = 0.14
R: 0.854
NR: -0.112
Corr=0.926
p val = 4.2e-08
R: 0.892
NR: 0.921
Corr=0.847
p val = 0.00019
R: 0.594
NR: 0.928
Corr=0.4
p val = 0.15
R: 0.535
NR: 0.052
Corr=0.521
p val = 0.14
R: 0.694
NR: 0.0879
Corr=0.779
p val = 0.0011
R: 0.502
NR: 0.889
Corr=0.267
p val = 0.49
R: 0.517
NR: -0.15
Corr=0.342
p val = 0.59
R: 0.662
NR: -0.141
Corr=0.27
p val = 0.55
R: 0.295
NR: -0.153
Corr=0.493
p val = 0.23
R: 0.576
NR: 0.00659
Corr=0.493
p val = 0.046
R: 0.434
NR: 0.487

Figure S4

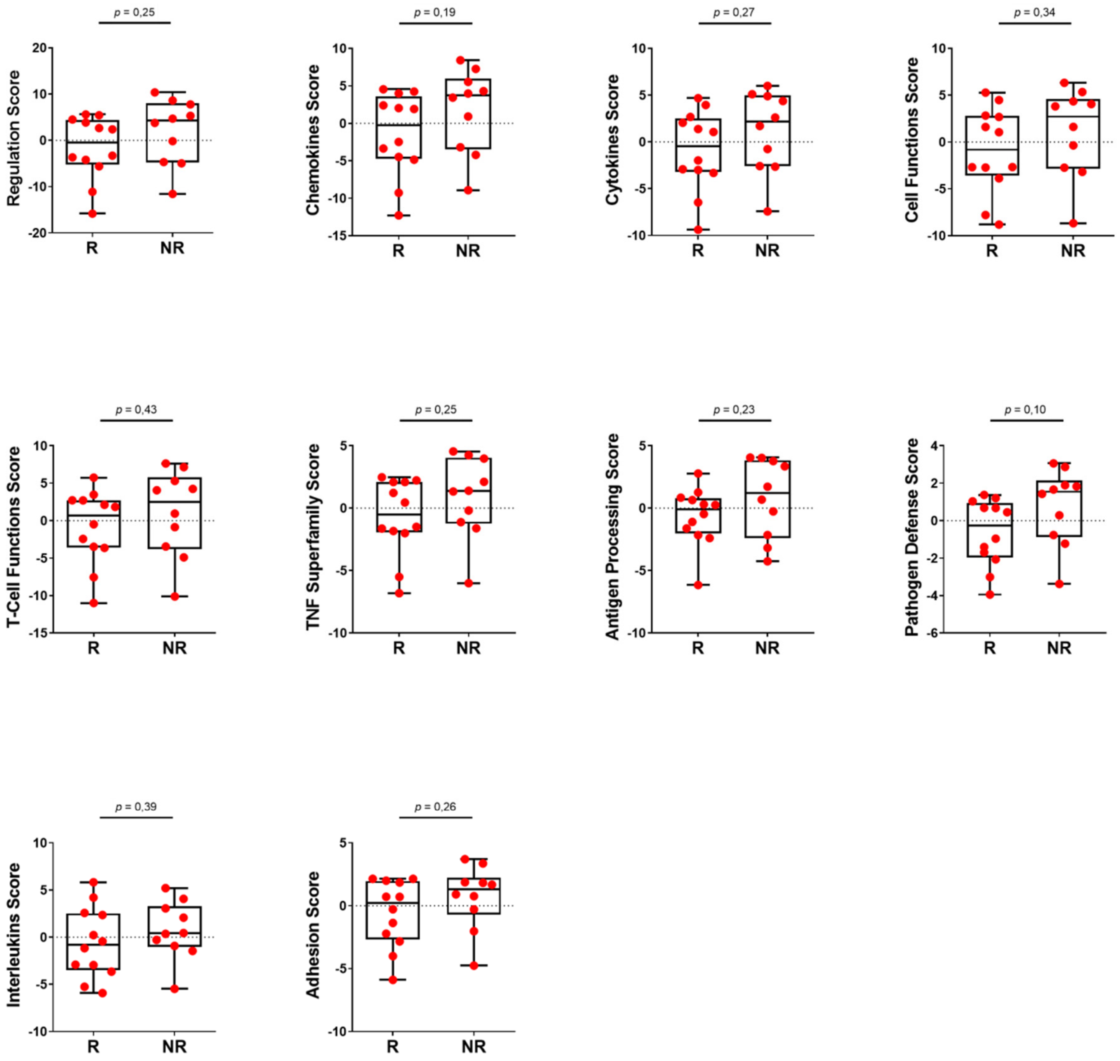
p = 0,25
Regulation Score
R
NR
p = 0,19
Chemokines Score
R
NR
p = 0,27
Cytokines Score
R
NR
p = 0,34
Cell Functions Score
R
NR
p = 0,43
T-Cell Functions Score
R
NR
p = 0,25
TNF Superfamily Score
R
NR
p = 0,23
Antigen Processing Score
R
NR
p = 0,10
Pathogen Defense Score
R
NR
p = 0,39
Interleukins Score
R
NR
p = 0,26
Adhesion Score
R
NR

Figure S5
